# A70 INABILITY TO AMBULATE FOLLOWING COLONOSCOPY: AN UNEXPECTED COMPLICATION

**DOI:** 10.1093/jcag/gwad061.070

**Published:** 2024-02-14

**Authors:** A Balbaa, E Brackenridge, M Tarnopolsky, M Sherlock

**Affiliations:** Pediatric Gastroenterology, McMaster University, Hamilton, ON, Canada; Pediatric Gastroenterology, McMaster University, Hamilton, ON, Canada; Pediatric Gastroenterology, McMaster University, Hamilton, ON, Canada; Pediatric Gastroenterology, McMaster University, Hamilton, ON, Canada

## Abstract

**Background:**

The peroneal nerve innervates the lower extremities. Compression injury has been documented in the adult patients following the lithotomy position perioperatively, but has rarely been described in pediatric patients following colonoscopy.

**Aims:**

To report a rare complication following colonoscopy and discuss possible aetiologies.

**Methods:**

A chart review of a post-colonoscopy peroneal nerve injury in a pediatric patient was conducted along with a literature review.

**Results:**

A 12 year old boy with ulcerative colitis underwent a colonoscopy for flare symptoms. He was in the left lateral decubitus position for 60 minutes. Immediately after the procedure, he experienced loss of sensation from the left knee to the plantar aspect of the foot, and left foot drop. He had no symptoms of weakness or paresthesia in the right lower extremity or the upper extremities. Prior to the procedure, he reported normal strength, function, and sensation of all extremities. He had no history of injury, joint inflammation or previous surgery affecting his left leg. He had no recent history of steroid use or systemic illness.

Two months post procedure he had some symptom improvement and could independently ambulate. He had normal range of motion, strength, and sensation in the upper extremities and right lower extremity. His left leg had 4/5 strength on dorsiflexion and decreased pinprick and light touch sensation. He had difficulty with heel-toe walking.

Reflexes were normal.

Left leg X-ray and spine MRI were normal. Electrodiagnostic evaluation supported a left common peroneal nerve injury, predominantly demyelinating in nature with minimal axonal contribution, likely secondary to compression around the fibular head during the time of the colonoscopy. He showed full recovery and returned to baseline by 7 months.

**Conclusions:**

Peroneal nerve injury post colonoscopy is very rare. A study focusing on pediatric colonoscopy found that 3 of 746 patients experienced postoperative neuropathies in their lower extremities. Procedure times were between 70-120 minutes. Presentations and recovery periods were variable, lasting several days to 3 months.

The mechanisms of this injury are unclear and felt unlikely to be secondary to positioning alone. It is speculated that BMI, procedure length, frog leg position and position changes, as well as systemic inflammation may be contributory. Factors such as hypothermia, dehydration, hypotension, hypoxia, and electrolyte disturbances may further place patients at risk. Awareness of this rare event and potential modifiable risk factors is important.

**Funding Agencies:**

None

